# Experimentally Derived Hansen Solubility Parameters as a Screening Tool for the Formulation of Amorphous Solid Dispersions

**DOI:** 10.1021/acsomega.6c05058

**Published:** 2026-06-22

**Authors:** Adaeze R. Osakwe, Mira T. N. Le, Jessica A. Bramhall, Vladislav V. Klepov, Jason J. Locklin

**Affiliations:** † Department of Chemistry, Franklin College of Arts and Sciences, 1355 University of Georgia, 140 Cedar Street, Athens, Georgia 30602, United States; ‡ New Materials Institute, University of Georgia, 220 Riverbend Road, Athens, Georgia 30602, United States

## Abstract

A major challenge in pharmaceutical development is the poor aqueous solubility of crystalline active pharmaceutical ingredients (APIs) generated during the API discovery. Amorphous solid dispersions (ASDs) improve solubility by molecularly dispersing amorphous APIs within a glassy polymer matrix; however, their utility is often constrained by limited API loading capacity and incomplete API release. To address these challenges, this study employs experimentally derived Hansen solubility parameters (HSP) as a predictive tool to assess polymer/API compatibility early in formulation development, rather than relying on conventional trial-and-error approaches. HSP values for ten commercially relevant ASD polymers and three APIs were determined using an experimental solubility screening method in conjunction with HSPiP software. Guided by these miscibility predictions, ASDs were prepared by materials-sparing hot-melt extrusion using only 2–7 g of API. A linear relationship was observed between the relative energy difference (RED) and the maximum amorphous API soluble (MADS), where lower RED values led to higher API loadings, up to 45%. Polymer/API interactions characterized using FTIR and DSC revealed that specific intermolecular interactions significantly influence dissolution behavior. Notably, the release of amorphous griseofulvin increased by up to 235% in ASD formulations exhibiting minimal intermolecular interactions compared to the crystalline API. Collectively, these results demonstrate the reliability of experimentally derived HSP for predicting ASD miscibility and API loading capacity, while complementary physicochemical characterization provides mechanistic insight into dissolution behavior. This integrated framework enables the rational design of high API-loaded, high-performance ASD formulations.

## Graphical abstract

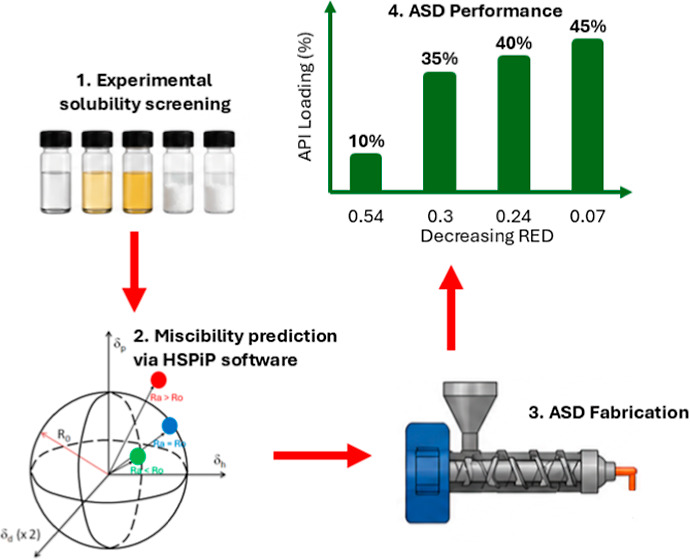

## Introduction

The amorphization of crystalline active pharmaceutical ingredients (API) has risen tremendously over the past three decades owing to the solubility limitations of orally dosed APIs in the pharmaceutical development pipeline. Rapid synthesis of API candidates enabled by combinatorial chemistry and high-throughput screening has led to 70% of APIs in the discovery phase being poorly soluble.^1^ API amorphization involves the conversion from its crystalline state, characterized by well-defined molecular structure and long-range order, to its amorphous form. Amorphous solids have higher molecular mobility, comparable to a liquid but with the viscosity of a solid, increasing their free energy relative to the crystalline counterpart.^2^ This enhances their solubility and bioavailability in oral solid dosage forms due to the disrupted molecular packing arrangement and weaker attractive forces between molecules.

An amorphous API is thermodynamically metastable and can revert to its crystalline form over a given time scale (seconds to years) based on the glass forming ability of the API. A matrix system, typically an amorphous polymer, can be employed to mitigate this instability, by molecularly dispersing the API to form an amorphous solid dispersion (ASD).^3^ However, high API loading increases recrystallization risk due to phase separation caused by molecular aggregation.^4,5^ Lowering API loading increases dosage quantity, which negatively affects patient compliance.^6^ The formulation of high-API load ASDs requires thermodynamic miscibility between API and polymer to ensure formation of a stable homogeneous mixture, with minimal risk of phase separation.^7^

Cohesive energy density (CED) and solubility parameters are key descriptors of miscibility between two molecules. The cohesive energy indicates the total attractive forces within a molecule, resulting from the summation of its intermolecular forces. Equation 1 details the relationship between CED and the solubility parameter. The Hansen solubility parameter (HSP) is most commonly used in literature to estimate polymer/API miscibility, loading capacity and stability of API in the formulation of ASDs.^8,9^ HSP can be calculated from group contribution (GC) methods, which estimates the property of a molecule based on summation of functional groups. However, this additive nature rests on the assumption that molecular properties are a sum of their parts, oversimplifying intermolecular interactions and electronic effects between functional groups, which reduces predictive accuracy for large, multifunctional molecules such as amphiphilic polymers commonly used in ASD formulations.^10,11^ Additionally, GC-based methods treat polymers as idealized repeat units and do not account for chain conformation, segmental mobility, or entropic contributions to mixing, all of which significantly influence polymer solubility and polymer/API miscibility in amorphous systems.^12,13^

1 $$\delta ={\left(CED\right)}^{0.5}$$

The limitations of predicting polymer/API miscibility using solubility parameters have been widely documented, with inconsistencies in GC-derived values identified as a major source of poor correlation with experimental ASD performance. Turpin et al. reported that solubility parameter differences and Flory–Huggins interaction parameters derived from GC methods often fail to reliably predict miscibility in ASD systems, particularly when strong hydrogen bonding or other specific interactions dominate.^12^

Consequently, experimental determination of solubility parameters has been recommended to improve predictive reliability. He and Ho specifically emphasized the importance of solvent-based experimental techniques to validate theoretical miscibility predictions during ASD development.^14^ Building on these recommendations, this work aims to bridge the gap between theoretical estimation of HSP values and experimentally derived miscibility predictions across a range of polymer/API systems.

HSP values of ten commercial ASD polymers were determined using empirical solubility data and HSPiP software. Four of these polymers, Eudragit EPO (EPO), Eudragit RLPO (RLPO), Kollidon VA 64 (KVA64), and HPMCAS L-grade (HPMCAS), were selected based on their distinct chemical functionalities and formulated with three APIs, carbamazepine (CBZ), griseofulvin (GRIS), and resveratrol (RSV), into ASDs via hot melt extrusion (HME) to evaluate the accuracy of the miscibility predictions. Thermal processing offers several advantages compared to solvent-based methods, including the elimination of solvent residues and the requirement of costly drying steps. Additionally, it has been used to make abuse-deterrent formulations, additive manufacturing for personalized medicine, and injection molding of solid dosage forms.^15,16^ Hence HME has received acclaim as a cost-effective and sustainable alternative for the continuous manufacturing of ASDs.

The versatility of HME is further emphasized in this study through the high melting temperatures (*T*_m_) of the three BCS class II model APIs, which prevents melting during processing. This allowed API/polymer miscibility, rather than melting behavior, to be the primary factor influencing API dissolution in polymer melt. It also addresses the limitation encountered when formulating ASDs of thermolabile APIs that degrade near their *T*_m_, as processing can occur well below melting. In this work, we demonstrate how miscibility predictions influence API loading capacity of each matrix, quantified as the maximum amorphous drug soluble (MADS). We hypothesize that API release behavior would have an inverse relationship with miscibility prediction, where poorly miscible pairs would exhibit faster release rates. However, our findings revealed that intermolecular interactions measured using spectroscopic analysis (FTIR) and deviation of glass transition temperature (*T*_g_) from ideal mixing behavior were the key determinants of dissolution performance.

## Experimental Section

### Materials

Eudragit EPO, Eudragit RLPO, Kollidon VA 64 and Soluplus were donated by BASF Corporation (Ludwigshafen, Germany). HPMCAS L, M, & H grades were donated by Shin-Etsu Chemicals (Tokyo, Japan). All HPMCAS grades used were of medium particle (MP) size. Povidone K30 was purchased from Spectrum Chemicals (New Brunswick, NJ, USA). HPC (Klucel MXF) and HPMC (Methocel K100M) were donated by Ashland Global (Wilmington, DE, USA). Resveratrol was purchased from Bulk Supplements (Henderson, NV, USA), carbamazepine was purchased from Thermo Fisher (Waltham, MA, USA), while Griseofulvin was procured from Astatech (Bristol, PA, USA). All the solvents for the solubility study were of ACS grades and above, purchased from commercial vendors and used as received.

### Polymer Solubility Analysis

The solubility of the polymers listed in Table 1 was conducted according to a revised method adapted from Patel et al. (2024) and Zellers et al. (1996).^18,19^ Polymer resins were melt-pressed into a film using a Carver press (Model 4386, Wabash, IN, USA), and punched into 12 mm diameter discs. Film thickness ranged from 150 to 350 μm, which depended on the melt viscosity of the polymer. Each polymer film (at a ratio of 10 mg of polymer per mL of solvent) was submerged in a 20 mL scintillation vial for 4 days. Ten organic solvents were initially selected, then additional solvents were subsequently included based on recommendation from the “solvent radius check” function of the HSPiP software. Observations were recorded after 1 and 24 h of polymer dissolution. After 96 h, undissolved polymers were withdrawn, blotted with a Kimwipe to remove residual solvent, and weighed to obtain the swollen mass (*m*_swollenmass_). Samples were then dried in a vacuum oven for 48 h at 40 °C to determine dry mass, *m*_drymass_. Normalized solvent uptake (*N*) was calculated with the solvent density, ρ_solvent_, as shown in eq 2.

2 $$N=1000\times \left(\frac{m_{swollenmass}-m_{drymass}}{m_{drymass}\times \rho_{solvent}}\right)\left(\frac{\mu L}{g}\right)$$

**Table 1:** Chemical Composition of Polymers

trade name	chemical composition
Eudragit EPO	poly(butyl methacrylate-*co*-(2-dimethylamino) ethyl methacrylate-*co*-methyl methacrylate). Comonomer ratio 1:2:1 ^17^
Eudragit RLPO	poly(ethyl acrylate, methyl methacrylate, 2-trimethylammonioethyl methacrylate chloride). Comonomer ratio 1:2:0.2 ^17^
HPC	hydroxypropylcellulose. *M*_w_ = 850 kDa
HPMC	hydroxypropylmethylcellulose
HPMCAS L-grade	hydroxypropylmethylcellulose acetate succinate. Methoxy DS (20–24%), hydroxypropoxy DS (5–9%), acetyl (5–9%), succinoyl (14–18%).
HPMCAS M-grade	hydroxypropylmethylcellulose acetate succinate. Methoxy DS (21–25%), hydroxypropoxy DS (5–9%), acetyl (7–11%), succinoyl (10–14%).
HPMCAS H-grade	hydroxypropylmethylcellulose acetate succinate. Methoxy DS (22–26%), hydroxypropoxy DS (6–10%), acetyl (10–14%), succinoyl (4–8%).
Kollidon VA 64	poly(vinylpyrrolidone-*co*-vinyl acetate). Comonomer ratio 60:40
Povidone K30	polyvinylpyrrolidone. *M*_w_ = 40 kDa
Soluplus	poly(vinyl caprolactam-polyvinyl acetate-polyethylene glycol) graft copolymer. Comonomer ratio 57:30:13

Based on the results, each polymer–solvent pair was assigned a score from 1 to 6. A score of 1 corresponds to the most soluble solvent, typically dissolving the polymer within an hour, while a score of 6 represents the least soluble solvent with the lowest *N* value (see Tables S1 and S2).

### API Solubility Study

A solution of each API was prepared in various organic solvents by incrementally adding 0.5 mL of solvent (up to 3 mL) to 5 mg of API. The scintillation vials were swirled at room temperature, then left to stand for 1 min, before additional solvent was added. This kinetic screening approach allows for measurement of a small molecule solubility over a concise period. Solvents that dissolved the API at 0.5 mL were assigned a score of 1, while those that failed to dissolve the API at 3 mL were given a score of 6. Details of solvent scores and scoring criteria are reported in Tables S1and S2.

### HSPiP Software

The assigned scores in Table S1 were used to obtain the Hansen solubility parameters using the solvent optimizer function in the HSPiP software. Each set was fitted using the Genetic Algorithm. More details are described in the Results and Discussion section.

### Polarized Optical Microscopy

Hot stage microscopy was used as prescreening tool to evaluate miscibility of each API with matrix polymers. Images were taken using an Eclipse LV100N POL (Nikon USA, USA) polarized optical microscope equipped with TMHS600 temperature-controlled stage (Linkam Scientific Instrument, UK). GRIS and RSV samples were heated from room temperature to 200 °C at 30 °C/min, while CBZ samples were heated to 170 °C. Samples were held at these temperatures for 10 min, with the application of slight pressure to reduce bubbles between glass discs, before cooling down to room temperature at the same rate.

### Hot Melt Extrusion

Powder mixtures were prepared by spatula mixing at incremental API loadings of 5% (w/w) until MADS was reached. The MADS is defined as the apparent maximum amorphous solubility of an API in a matrix, measured as the peak concentration where no defined crystalline API Bragg peaks are observed via X-ray diffractometry (XRD), see Figure S11. Polymer/API mixtures were processed using a Haake Minilab II conical twin screw extruder (Thermo-Fischer, USA) rotating at 100 rpm and cycled for 5 min before flushing. Each mixture contained 6 g of polymer, while API mass varied based on percent composition. API Processing temperatures for each polymer were optimized based on extrusion torque, outlined in Table 4.

### Thermogravimetric Analysis

Thermal degradation studies of APIs, excipients, and extrudates were conducted on a Discovery TGA550 (TA Instruments, USA). Masses between 7 and 12 mg of each sample were heated in a platinum pan from 30 to 600 °C at a rate of 10 °C per minute, under a nitrogen purge of 25 mL/min.

### Differential Scanning Calorimetry

DSC analysis of APIs, excipients, and extrudates was conducted on a Discovery DSC250 equipped with a RCS90 cooling system (TA Instruments, USA). Samples between 3 and 5 mg were crimped in aluminum Tzero pans and analyzed under a 50 mL/min nitrogen purge. The nonhermetic sealing allowed escape of volatiles, such as moisture during heating ramps. Pristine polymers and ASD extrudates were heated from −20 to 200 °C at 10 °C/min to erase thermal history and remove moisture due to the hygroscopic nature of the excipients. This was followed by cooling to −20 °C, and a subsequent heating ramp to 200 °C at the same rate. The *T*_g_s of CBZ and GRIS were obtained by heating the amorphous API (prepared by rapidly quenching from melt with liquid nitrogen) from −60 to 210 °C and 260 °C respectively at 10 °C/min.

### X-ray Diffractometry

Crystallinity was measured using powder X-ray diffraction (PXRD) on a Bruker D2 PHASER diffractometer using Cu Kα radiation over a 2θ range 5–23° with a step size of 0.02° on a silicon slide. Pure API and excipients were characterized in their pristine powdered state, while the extrudates were ground cryogenically using a CG-500 Cole-Parmer (Antylia Scientific, USA) freezer mill. Characterization of extrudates to determine MADS was carried out 1 week post extrusion. Samples were stored under ambient conditions on a laboratory bench at approximately 22 °C and 50% RH.

### Fourier Transform Infrared Spectroscopy

A 6700 Nicolet FTIR equipped with a GATR (Thermo Fisher Scientific, Waltham, MA, USA) was used for FTIR spectroscopic characterization of all APIs, polymers, and extrudates. Each analysis was from an average of 64 scans between 4000 and 400 cm^–1^, with a resolution of 0.482 cm^–1^.

### In Vitro Dissolution Study

All dissolution testing was conducted in triplicates using a Distek 2500 system (North Brunswick, NJ, USA) setup according to USP apparatus 2. Powderized extrudates and neat API were dispersed in vessels containing 500 mL of 0.05 M phosphate buffer, pH 6.8 at 37 °C, with a paddle speed of 100 rpm. A 1 mL aliquot was collected, without replacement, at *t* = 5, 15, 30, 45, 60, 90, and 120 min, then filtered with a 0.20 μm PTFE membrane filter for HPLC analysis.

### High Performance Liquid Chromatography

Detection and quantification for all APIs were conducted using an Agilent 1100 Series (Agilent, USA) HPLC system equipped with a ZORBAX Eclipse C18 (150 mm × 4.6 mm with 5 μm particle size) column (Agilent, USA). Details of the chromatographic conditions of each API are outlined in the Table S3.

## Results and Discussion

### Solubility Study and HSP Computation

Polymers displayed distinct solvation profiles in different solvents based on the chemical structures, physicochemical properties, and thermodynamic compatibility of polymer and solvent. For certain polymers, addition of solvent led to dissolution within minutes, while others showed minimal swelling upon 96 h exposure to solvent. Each solvent was assigned a score between 1 and 6 based on its relative solvency behavior. The scoring criteria are outlined in Table S2. For both polymers and APIs, solvents with a score of 1 were designated as “inside” solvents on the HSPiP software—that is, solvents contained within the solubility sphere of the polymer in the 3D Hansen space. Solvents with scores from 2 to 6 fall outside the sphere, with higher scores corresponding to greater radial distance from the sphere center, therefore decreasing polymer–solvent compatibility.

The HSPs of each polymer and API were subsequently determined using the genetic algorithm (GA) optimization function implemented in HSPiP. This algorithm identifies the smallest possible solubility radius (*R*_0_), while iteratively refining the sphere position to best separate good and poor solvents. Unlike the classic Hansen algorithm which penalizes near-boundary solvents excessively, the GA fitting preferentially places the “least wrong” solvents near the boundary, thereby accommodating rare cases where outside solvents exhibit partial swelling behavior.^20^ When poor solvents are assigned scores that places them within the solubility sphere, or good solvents are placed outside the sphere, the Fit value decreases. The experimental Fit, reported as a fraction, indicates the accuracy of classifying good solvents inside the sphere or poor solvents outside sphere. In this work, the Fit value for all polymers and API characterized have a Fit value of 1.

The experimentally determined HSP of each polymer and API are reported in Table 2. The total HSP, or total cohesive energy density of the molecule, is made up of three components: dispersion (δ_D_), polarity (δ_P_), and hydrogen bonding (δ_H_). These parameters represent the three coordinates of the 3D sphere in the Hansen space. The magnitude of each component reflects its relative contribution to the total cohesive energy of the molecule, δ_total_, as expressed in eq 3.

3 $$\delta_{total}^{2}=\delta_{D}^{2}+\delta_{P}^{2}+\delta_{H}^{2}$$

**Table 2:** Experimentally Determined HSP Values of Ten ASD Excipients and Three Model APIs

polymer/API	δ_D_	δ_P_	δ_H_	Δ_total_
Eudragit EPO	17.2	5.3	8.6	19.9
Eudragit RLPO	17.4	9.2	8.6	21.5
HPC	18.4	12.4	8.4	23.7
HPMC	18.3	6.6	10.5	22.2
HPMCAS L-grade	17.4	10.4	9.2	22.3
HPMCAS M-grade	17.1	12.6	6.7	22.3
HPMCAS H-grade	16.5	8.8	7.5	20.1
Kollidon VA 64	16.5	10.2	10.4	22
Povidone K30	16.9	9.3	11.3	22.3
Soluplus	17.4	10	4.9	20.7
carbamazepine	16.5	9.8	10.3	21.7
griseofulvin	17.2	10.7	8.4	21.9
resveratrol	15.9	10.9	9.7	21.6

### ASD Formulation and Miscibility Calculation

From the ten polymers in Table 2, four representative polymers (Eudragit EPO, Eudragit RLPO, HPMCAS (L-grade) and KVA64) were selected for subsequent ASD formulation and miscibility analyses with the model APIs. These polymers, shown in Figure 1, possess diverse chemical functionalities: Eudragits are methacrylate-based, HPMCAS is a cellulosic derivative, and KVA64 is pyrrolidone-based. Two methacrylate-based polymers were selected (EPO and RLPO) to assess how moderate differences in chemical functionality influence polymer/API miscibility.

**Figure 1:**
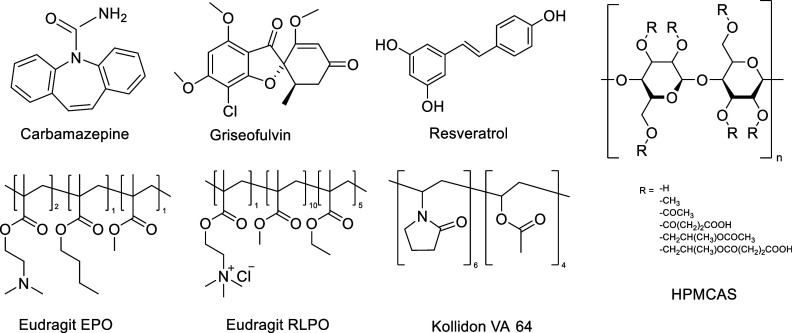
Chemical structures of APIs and polymers used in miscibility study.

Polymer/API miscibility was evaluated using the relative energy difference (RED), defined as the ratio of the Hansen distance between the two components (*R*_a_) to the polymer’s solubility radius (*R*_0_) (see eqs 4 and 5). An RED of zero indicates ideal compatibility, whereas values greater than one suggest immiscibility.^21^ Table 3 shows that all 12 polymer/API pairs in this study exhibited RED <1, indicating thermodynamic compatibility to varying extents. For instance, carbamazepine had RED values of 0.07 with KVA64 and 0.54 with EPO, implying greater miscibility, and likely a higher amorphous solubility limit, in KVA64 than in EPO.

4 $${\left(R_{a}\right)}^{2}=4{\left(\delta_{D2}-\delta_{D1}\right)}^{2}+{\left(\delta_{P2}-\delta_{P1}\right)}^{2}+{\left(\delta_{H2}-\delta_{H1}\right)}^{2}$$

5 $$RED=\frac{R_{a}}{R_{0}}$$

where: 1 = polymer, and 2 = drug.

**Table 3:** RED Values of Each Polymer/API Combination

	Eudragit EPO	Eudragit RLPO	HPMCAS	Kollidon VA 64
carbamazepine	0.54	0.30	0.24	0.07
griseofulvin	0.57	0.19	0.16	0.20
resveratrol	0.68	0.41	0.38	0.21

On average, EPO exhibited the highest RED with the model APIs, while KVA64 showed the lowest, with RLPO and HPMCAS displaying intermediary values. This trend is consistent with the molecular features and HSP characteristics of EPO (Table 2), which indicate a comparatively low degree of polarity relative to the other polymers. The diminished polar interactions reduce EPO’s affinity for the more polar APIs, leading to poorer polymer/API miscibility; consequently, reduced thermodynamic stability and amorphous solubility of APIs within ASD formulations.

### Hot Melt Extrusion

To ensure that API amorphization was driven primarily by polymer/API miscibility rather than by API melting, polymers were processed at temperatures substantially below the melting points of each API. This approach is particularly critical for thermally labile compounds such as resveratrol, for which melting and the onset of thermal degradation occur simultaneously.

Table 4 displays the glass transition temperature for each polymer along with the extrusion temperature used. The processing temperatures were optimized using the melt viscosities of the neat polymers and manufacturer-recommended processing ranges. Across the 12 polymer/API combinations evaluated, torque values (a measure of melt viscosity) were maintained within 0.5–1 N·m. Increasing the processing temperature above the selected range did not lead to an increase in the MADS values for any polymer. In contrast, a reduction in temperature led to high melt viscosity, causing the torque to approach the upper limit of the instrument (2.0 N·m) and prohibit further processing.

**Table 4:** Optimization of Processing Temperature for Each Polymer

polymer	glass transition (°C)	extrusion temperature (°C)
Eudragit EPO	47	150
Eudragit RLPO	53	160
HPMCAS	123	170
Kollidon VA 64	110	160

The stability of the extrudates was analyzed with thermogravimetric analysis (TGA), results shown in Figures S1–S3. The APIs, polymers and ASDs (up to 10 w/w %), were observed to be thermally stable well above the processing temperatures of each polymer.

#### API Amorphization

Figure 2 displays the PXRD diffractograms for each API at the MADS value within the corresponding polymer matrix. Crystallinity in the ASDs was assessed by analyzing HME extrudates prepared at 5 wt % increments until saturation of the polymer matrix was observed. The appearance of defined sharp Bragg peaks in the diffractograms indicated presence of excess crystalline API and its phase separation, see Figure S11. Extrudates were processed at temperatures lower than those used for POM screening (Table 4), since the shear mixing imparted during HME promotes API dissolution at reduced temperatures. Figure 2a indicates that KVA64 has the highest capacity to dissolve carbamazepine with a MADS value of 45%, HPMCAS (40%), RLPO (35%), while EPO exhibits the lowest capacity at 10%; MADS values decreased with corresponding increase in RED, see Table 3. For griseofulvin (Figure 2b), comparable MADS values of 15% were observed in HPMCAS, RLPO and KVA64, which correspond to RED values of 0.16, 0.19, and 0.20 respectively. In contrast, a MADS of only 5% was observed with EPO, which is consistent with the RED value of 0.57. Finally, resveratrol (Figure 2c) exhibited the highest solubility in KVA64 with a MADS of 35%, similar to carbamazepine and consistent with POM observations. Lower MADS values were obtained with RLPO (20%), HPMCAS (15%) and EPO (10%). The reported MADS values represent apparent amorphous solubility limits, observed over the specific processing and storage conditions employed in this work. While long-term stability data are provided (Figure S9), HSP does not predict kinetic stability or time-dependent recrystallization behavior of an API. Therefore, the MADS values reported here are intended as experimentally observed screening metrics rather than equilibrium thermodynamic solubility values.

**Figure 2:**
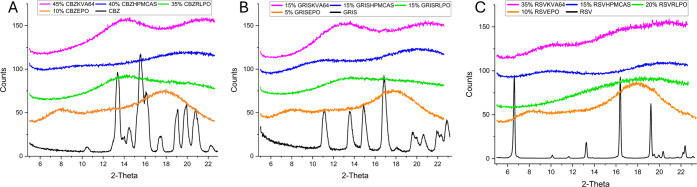
X-ray diffractograms of ASDs containing (A) carbamazepine, (B) griseofulvin and (C) resveratrol that shows the maximum amorphous API soluble in each polymer matrix. The diffractogram of the crystalline API is also shown in black.

In agreement with our proposed hypothesis, Figure 3 demonstrates that MADS in ASD extrudates exhibited an inverse linear correlation with the RED values predicted using experimental HSP. In contrast, no correlation was observed between MADS values and RED values calculated using group contribution HSP. Group contribution methods determine HSP by summing up energy contributions from constituent functional groups.^22^ However, this approach does not account for factors such as functional group position, intramolecular interactions, and molecular weight.^23^ This results in inconsistency when used to predict miscibility of complex ASD systems composed of high molecular weight polymers and APIs with varying degrees of amphiphilicity.

**Figure 3:**
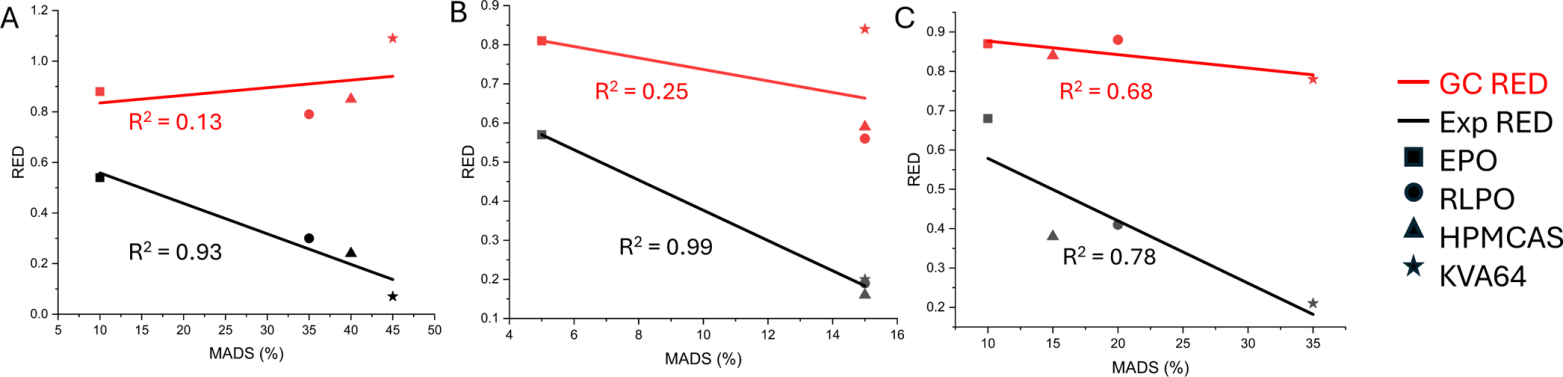
Regression analysis of (A) carbamazepine, (B) griseofulvin, and (C) resveratrol MADS in each matrix with RED derived from experimental (black) and group contribution (red) HSP calculations.

Due to the highly conjugated nature of its stilbene structure and the presence of three hydroxyl groups, resveratrol has a high tendency to self-assemble^24^ compared to the other two model APIs used in this study. Resveratrol undergoes rapid crystallization with high tendency to form nanocrystals,^25^ hence, inhibiting miscibility with polymers. This is theorized to be the cause of the discrepancy in the *R* ^2^ values seen in Figure 3. In contrast, griseofulvin with an *R* ^2^ of 0.99 is a rigid spirocyclic molecule with a single aromatic ring that is heavily substituted, and a lack of hydrogen bond donors. This structural difference is key in preventing nanocrystal formation, and maintaining molecular similarity measured via HSP as the key driving factor in predicting miscibility and amorphous API solubility.

The MADS values obtained in this work show good agreement, and in some cases exceed previously reported amorphous solubility limits for these APIs. While solvent-based methods such as spray drying achieve higher API loadings due to advantages in viscosity and rapid solvent evaporation, they are also frequently associated with greater thermodynamic instability.^26–28^ Bennett et al. reported griseofulvin to exceed its amorphous solubility in Eudragit L100–55 at 25%;^29^ while in HPMCAS, the maximum amorphous solubility was reported to be 10% when processed via hot melt extrusion.^30^

Similarly, carbamazepine has been reported to exceed its amorphous solubility when extruded with KVA64 at 25%,^31^ lower than values reported in this work. In HPMCAS, 30% amorphous loading was achieved in a ternary blend containing 70% Soluplus.^32^ In another study using KVA64/EPO at a 1:2 ratio, saturation of CBZ occurred at 15%,^33^ which aligns well with our MADS value of 10% in EPO and 45% KVA64.

In investigating the chemical stability of resveratrol, Zhou et al.^34^ observed 20% RSV to be amorphous in a binary extrudate with HPMCAS when processed at 210 °C, but crystalline at 140 °C. Although comparable amorphous solubility limits were attained, their processing temperature was 40 °C higher than that employed in this study. In a spray dried solid dispersion with PVP, 35%, resveratrol was observed to remain amorphous, while HPMCAS recrystallized with 1 h at the same concentration,^35^ comparable to observations seen in this work with KVA64.

### Polarized Optical Microscopy

Polarized optical microscopy (POM) with a hot-stage heating attachment was evaluated as a materials sparing prescreening tool to assess polymer/API miscibility behavior. Under crossed polarizers, birefringent domains indicate the presence of undissolved crystalline API, whereas loss of birefringence indicates API dissolution or amorphization within the polymer melt.^36^ Mixtures of CBZ with each polymer were heated to 170 °C, while RSV- and GRIS-polymer mixtures were heated to 200 °C. These temperatures were selected to enable the observation of API solubilization above the softening points of each polymer matrix, while remaining below the melting point of each API. The experimental temperature of RSV and GRIS was increased to 200 °C to reduce the experimental time scale, while maintaining temperatures below their respective *T*_m_ values.

CBZ exhibits polymorphism, with four known stable anhydrous forms. Form III, which has a prismatic morphology and is commonly found in commercial samples, melts at 173 °C. This polymorph is metastable relative to the higher melting Form I, which exhibits needle-like crystals and melts at 191 °C.^37^ Upon heating mixtures containing CBZ at its MADS concentration, crystalline domains disappeared at 170 °C, consistent with dissolution into the polymer melt and transformation to its amorphous state. This behavior suggests favorable polymer/API interactions capable of disrupting the CBZ crystal lattice leading to its melting. In contrast, when CBZ concentrations exceeded the experimentally determined MADS, undissolved prismatic crystals persisted (Figure 4a–f), indicating a saturation point in the glassy polymer matrix, where no additional API can be incorporated in the amorphous state. The observed MADS concentration for CBZ in each matrix is 10% in EPO, 35% in RLPO, and 45% in KVA64.

**Figure 4:**
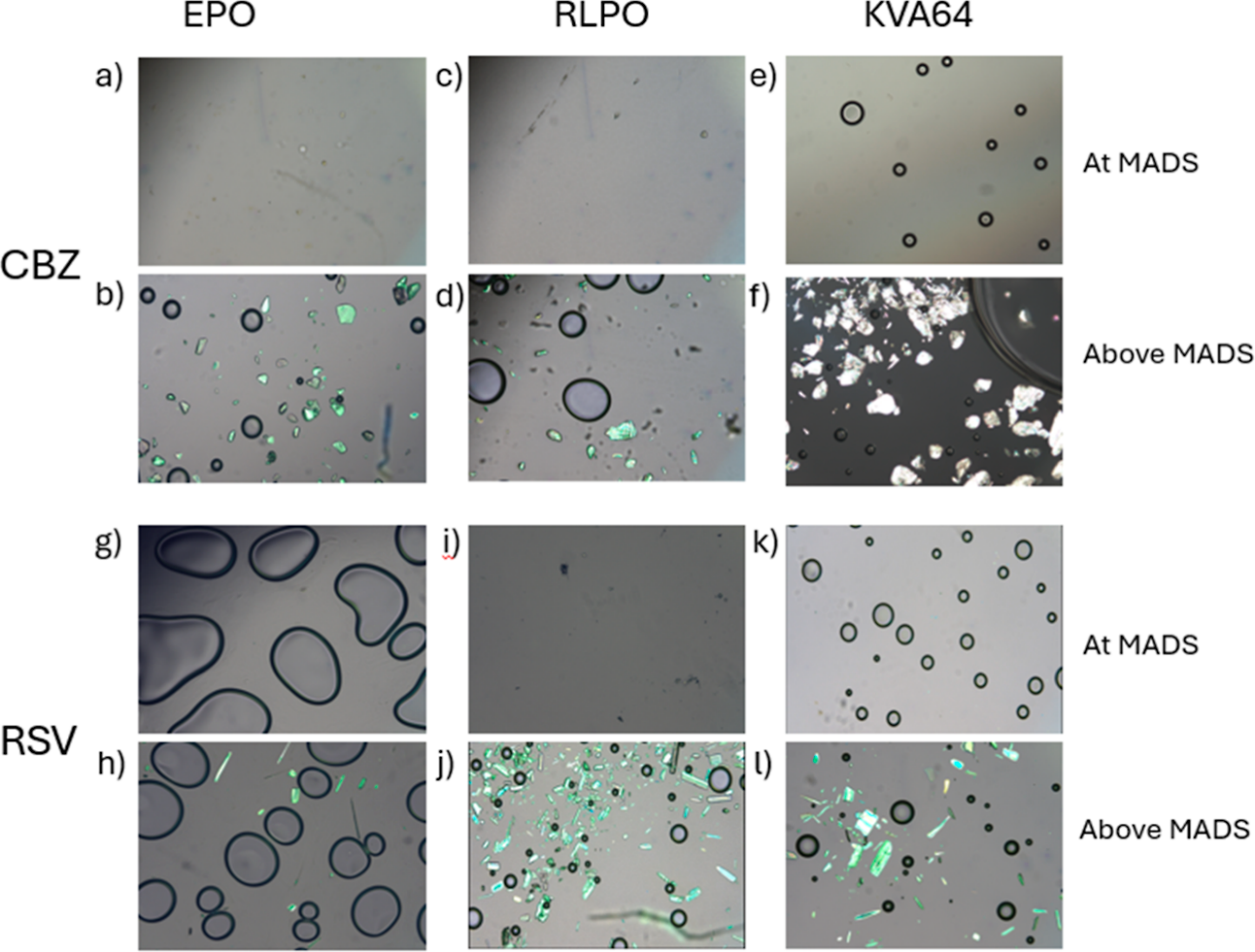
Polarized optical micrographs of physical mixtures of at CBZ and RSV in three different glassy polymer matrices at and above the maximum amorphous API soluble concentration. (a) 10% CBZ in EPO, (b) 15% CBZ in EPO, (c) 35% CBZ in RLPO, (d) 45% CBZ in RLPO, (e) 50% CBZ in KVA64, (f) 50% CBZ in KVA64 (g) 10% RSV in EPO (h) 15% RSV in EPO (i) 20% RSV in RLPO (j) 25% RSV in RLPO (k) 35% RSV in KVA64 (l) 40% RSV in KVA64. The bubbles in some of the images are due to entrapped air between the two coverslip slides.

For RSV, which has a higher melting temperature at 288 °C and strong tendency to rapidly crystallize,^25^ miscibility assessments were carried at 200 °C, well below its *T*_m_. Similar trends to CBZ were observed, where needle-like birefringent crystals persisted above polymer-specific concentration thresholds, indicative of undissolved RSV and defining its maximum solubility in each matrix (Figure 4h–l). KVA64 dissolved the highest concentration of RSV at 35%, followed by RLPO at 20%, while EPO dissolved the least concentration at 10%. This correlates with the smaller RED value of KVA64 to RSV at 0.21, RLPO at 0.41, and EPO at 0.68, see Table 3.

The utility of POM as a prescreening tool was limited with its application to GRIS and HPMCAS. Although the glass-forming ability (GFA) of GRIS is categorized as Class 1 when cooled at 20 °C/min under the classification of GFA molecules by Baird et al.;^38^ however, its molecular weight of 352 g/mol exceeds the 300 g/mol threshold above which pharmaceutical molecules have a stronger tendency to form more stable glasses.^39^ This contributes to the relatively high amorphous stability of GRIS,^40^ allowing partial amorphization to occur under mild heating and resulting in poorly defined birefringence, even at elevated API loadings.^41^ Consequently, crystalline domains become less distinguishable under crossed polarizers, reducing the sensitivity of POM for assessing GRIS-polymer miscibility, see Figure S4i. Additionally, HPMCAS was excluded from the POM screening due to the presence of birefringent cellulose crystallites in the neat polymer, which interfered with distinguishing API crystals before and after complete amorphization (Figure S4).

#### Polymer/API Interactions

In this work, FTIR spectroscopy and deviations from ideal mixing behavior, observed through the *T*_g_ values of individual components and the ASDs, were used to qualitatively identify polymer/API interactions. These interactions can constitute a significant limitation to dissolution even at low API concentrations. The formation of hydrogen bonding interactions, ionic complexes, and other noncovalent associations between the API and polymer matrix has been shown to restrict release and reduce apparent dissolution performance.^42–44^

### Carbamazepine

Understanding polymer/API interactions is crucial in the formulation of amorphous solid dispersions.^42–45^ Carbamazepine is a weakly basic API, where the carbamide moiety can act as a proton donor and acceptor. The nitrogen on the azepine ring and primary amide can interact with the electron withdrawing substituents of each polymer. Figure 5 examines the FTIR spectra of the crystalline and amorphous form of CBZ, along with ASDs with increasing loadings (up to MADS) in EPO (5a), RLPO (5b), and HPMCAS (5c). The C=O stretch in crystalline CBZ is assigned to 1673 cm^–1^ and the peaks at 1604 cm^–1^ and 1593 cm^–1^ represent the C=C stretch and N–H deformation.^37^ These peaks broaden slightly in the amorphous form generated from rapidly quenching CBZ from the melt in liquid N_2_. The C=O stretch of CBZ shifts to higher wavenumber in each polymer matrix to varying degrees (1687 cm^–1^ in EPO, 1682 cm^–1^ in RLPO, and 1680 cm^–1^ in HPMCAS). The blueshifts can be attributed to a delocalization of the carbonyl bond, likely due to hydrogen bond formation between CBZ amide and the polymer backbone. An increase in wavenumber of the CBZ carbonyl peak in CBZ-EPO was also observed by Feng et al., differing from the physical mixture of the API and the polymer, where no peak shifts were observed.^46^ With KVA64 (see Figure S10), minimal changes are observed on CBZ carbonyl peak, which closely overlaps with the pyrrolidone carbonyl of the polymer. This result also supports a previous study by Yu et al., where minimal interactions between PVPVA and CBZ are observed in both experimental and molecular modeling studies.^47^ HPMCAS presents a new peak at 1708 cm^–1^, likely due to interactions between CBZ carbonyl and the COOH of the succinyl group in HPMCAS, thereby disrupting of CBZ–CBZ intermolecular interactions, consistent with reports from Ishizuka et al.^28^ Similarly, a new peak is observed on the CBZ carbonyl speak at about 1650 cm^–1^, likely due to hydrogen bonding interactions between CBZ C=O and HPMCAS.^28^

**Figure 5:**
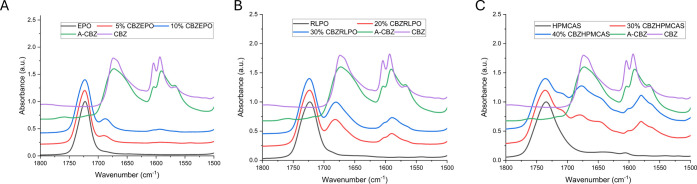
FTIR spectra of carbamazepine (crystalline and amorphous form) and the ASDs formed with (A) EPO, (B) RLPO, and (C) HPMCAS. ASD matrices displayed in each graph are at and below MADS concentration.

With respect to the C=C stretch and N–H bending vibrations in CBZ, the coalescence of the crystalline doublet at 1604 cm^–1^ and 1595 cm^–1^ in amorphous CBZ, indicates the loss of ordered lattice interactions and formation of the amorphous form.^48^ This is also observed in each ASD matrix made using HME, confirming the conversion from crystalline to amorphous API.

### Griseofulvin

Figure 6 shows the FTIR of griseofulvin incorporated within three polymer matrices, EPO (6A), RLPO (6B), and HPMCAS (6C). The crystalline form of griseofulvin has three characteristic peaks at 1615, 1598, and 1584 cm^–1^ corresponding to the aromatic C=C stretching and C=C–O conjugated vibrations within the benzofuran and cyclohexenone ring systems. Upon amorphization from rapid quenching, these distinct peaks merge into broader peaks centered at 1607 and 1583 cm^–1^. The broadening of these bands is indicative of reduced vibrational coupling in the aromatic rings and conjugated carbonyls, characteristic of the amorphous solid of GRIS.^49^ These broad peaks are also observed in each polymer matrix made using HME at and below MADS, which is evidence for successful amorphization of the API in each polymer matrix.

**Figure 6:**
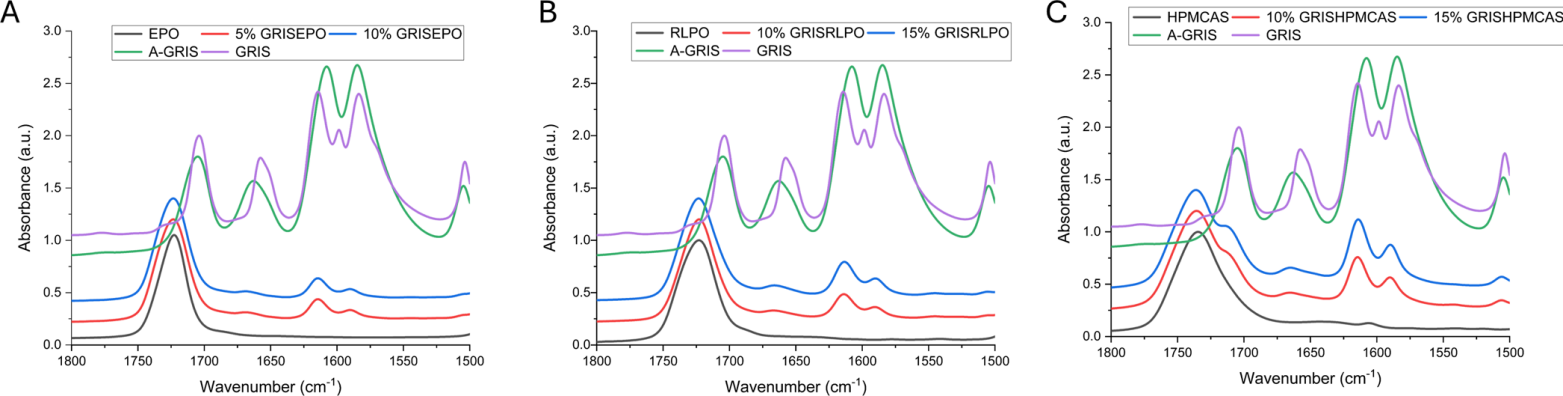
FTIR spectra of griseofulvin (crystalline and amorphous form) and the ASDs formed with (A) EPO, (B) RLPO, and (C) HPMCAS. ASD matrices displayed in each graph are at and below MADS concentration.

In the crystalline form, griseofulvin exhibits two prominent carbonyl stretching vibrations associated with the benzofuran lactone and the cyclohexenone observed at 1705 cm^–1^ and 1657 cm^–1^ respectively. Upon amorphization by rapid quenching, the cyclohexenone carbonyl broadens and shifts to higher wavenumber (1663 cm^–1^). This shift is apparent in each polymer matrix, varying at 1669 cm^–1^ in EPO, and 1665 cm^–1^ for both RLPO and HPMCAS. Previous studies attribute this peak broadening to the formation of intermolecular hydrogen bonds between HPMCAS-L and GRIS.^26,50^ In contrast, the benzofuran carbonyl stretch (1705 cm^–1^), remains unchanged in the crystalline and amorphous form of GRIS, but increases in the ASDs. In HPMCAS, the band shifts to 1712 cm^–1^, while in EPO and RLPO there is a stronger blueshift leading to an overlap with the polymer carbonyl absorption centered at 1724 cm^–1^.

### Resveratrol

The characteristic C=C stretching vibrations of the conjugated aromatic rings in crystalline resveratrol appear at 1583 and 1605 cm^–1^. These two peaks coalesce and broaden, reflecting the loss of long-range orders in the ASDs. Amorphization of pure resveratrol could not be achieved through quench cooling due to its thermolability and narrow processing window between its melting (278 °C) and degradation temperature (283 °C).

In Figure 7, the carbonyl bands of each polymer matrix (RLPO, HMPCAS, and KVA64) are centered around ∼1725 cm^–1^. The C=O stretch in RLPO ASDs (Figure 7a) display a shoulder at lower wavenumber, which is likely due to hydrogen bonding with the phenolic hydroxyls of resveratrol. With respect to HPMCAS (Figure 7b), minimal change in the carbonyl stretch is observed. In contrast, the KVA64 pyrrolidone carbonyl component at 1655 cm^–1^ (Figure 7c) shifts to 1649 cm^–1^ due to hydrogen bonding with RSV.

**Figure 7:**
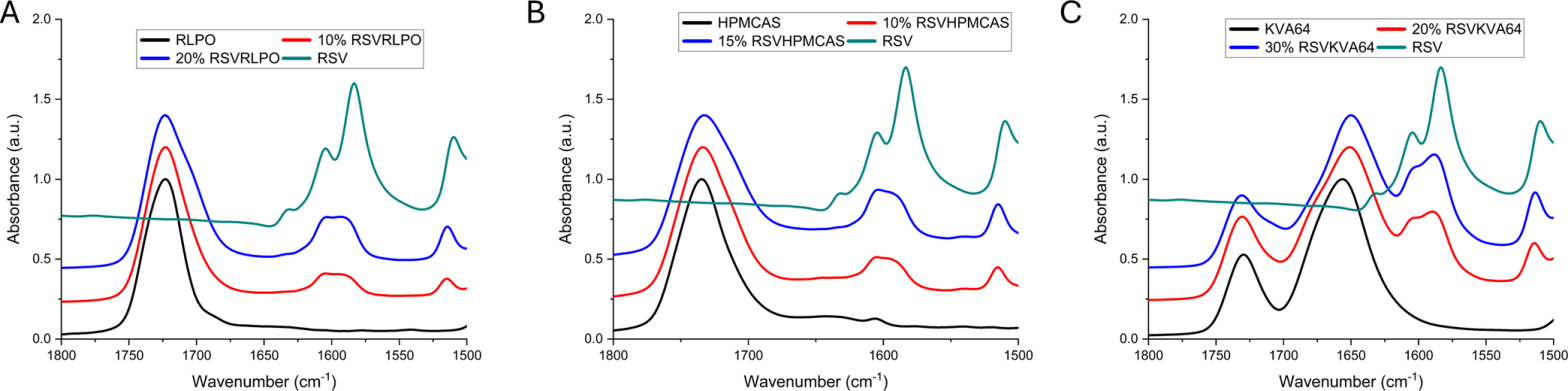
FTIR spectra of resveratrol (crystalline form) and the ASDs formed (A) RLPO, (B) HPMCAS, and (C) KVA64. ASD matrices displayed in each graph are at and below MADS concentration.

### Gordon-Taylor Model

The Gordon-Taylor equation was employed to evaluate deviations from ideal mixing behavior between the API and polymer matrix. Equation 6 provides a theoretical prediction of the glass transition temperature (*T*_g_, calc) for a binary system based on the weight fractions (*w*_1_ and *w*_2_) and glass transition temperature of each component (*T*_g1_ and *T*_g2_)­

6 $$T_{g},calc=\frac{w_{1}T_{g1}+Kw_{2}T_{g2}}{w_{1}+Kw_{2}}$$

*K* is a constant that can be calculated from eq 7, which relies on the true densities (ρ_1_, ρ_2_) and change in thermal expansivity across *T*_g_ (Δα_1_, Δα_2_) of each component. These values were experimentally determined using a helium pycnometer and modulated DSC (Table S4) with the assumption that the change in heat capacity is proportional to the change in thermal expansion across *T*_g_.^51^

7 $$K=\frac{\rho_{1}{\Delta \alpha }_{2}}{\rho_{2}{\Delta \alpha }_{1}}$$

A positive deviation between the experimentally measured *T*_g_ (*T*_g_,_exp_) and Gordon-Taylor estimated *T*_g_ (*T*_g_,_calc_), whereby *T*_g_,_exp_ > *T*_g_,_calc_, indicates antiplasticization of the polymer matrix by the API. This restricts molecular mobility and reduces segmental motion. The resulting increase in chain rigidity limits polymer relaxation upon exposure to water and can hinder API release during dissolution.^52^ Conversely, when *T*_g_,_exp_ < *T*_g_,_calc_, the system exhibits plasticization, reflecting increased chain flexibility and free volume induced by the API. This enhances the ability of the polymer to absorb water and facilitates API release, in the absence of specific intermolecular interactions.^53^

In Figure 8A, carbamazepine shows near ideal mixing in the KVA64 matrix as the experimental and theoretical *T*_g_s overlap. RLPO shows a positive deviation at low concentrations but begins to approach ideal mixing at higher concentrations. A negative deviation from ideal mixing is observed in both HPMCAS and EPO matrices. Therefore, CBZ can plasticize both matrices, enhancing chain mobility and processability at lower temperatures.

**Figure 8:**
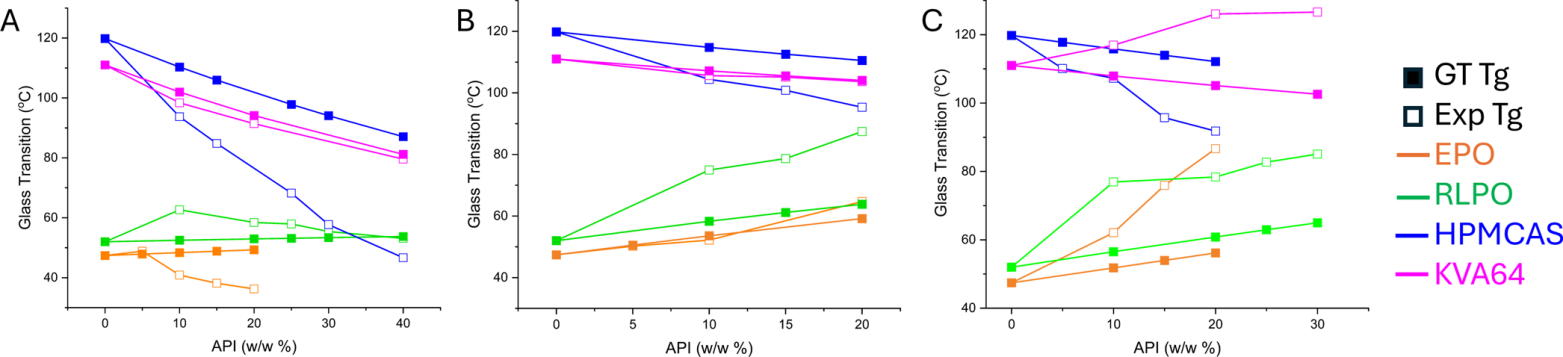
Graphs showing experimental glass transition (open squares) of (A) carbamazepine, (B) griseofulvin, and (C) resveratrol deviating from ideal behavior predicted using Gordon Taylor equation (filled squares). The DSC thermograms used to generate the plots are provided in the (Figures S5–S8).

In Figure 8B, GRIS maintains ideal mixing behavior with KVA64 and EPO, with EPO showing a slight positive deviation at the highest concentration tested. RLPO reveals a positive deviation from ideal mixing, while HPMCAS displays a negative deviation from ideal mixing.

In Figure 8C, resveratrol exhibited a positive deviation from ideal mixing behavior with EPO, RPLO, and KVA64 matrices, while HPMCAS matrix was plasticized by the API. FTIR analysis indicates hydrogen bonding between the phenolic hydroxyl groups of RSV and polymer carbonyls in EPO, RLPO and HPMCAS (Figures 7A,C and S10C). However, the plasticized HPMCAS system shows comparatively weaker interactions (Figure 7B). These differences significantly impact the dissolution behavior of RSV.

Overall, the *T*_g_ trends demonstrate that an API can act as either a plasticizer or antiplasticizer depending on the polymer matrix. No clear correlation was observed between the magnitude of the RED values (Table 3) and either the strength of interactions by FTIR or the direction of deviation from Gordon-Taylor predicted *T*_g_. This highlights a key limitation of HSP-based approaches in capturing performance of complex systems, particularly with copolymers and multifunctional APIs.

#### API Dissolution Studies

In vitro dissolution experiments were performed under sink conditions to ensure release was not limited by the aqueous solubility of each API. Each ASD and corresponding crystalline API was evaluated at a fixed API concentration of 20 μg/mL in the dissolution medium. Factors such as API/water and polymer/water interactions have also been shown to limit drug release under certain conditions.^45,54^ However, these effects are typically compounded at high API loadings, where phase separation occurs and amorphous API forms hydrophobic domains that hinder polymer dissolution.^55,56^ Consequently, a low API loading of 10% was selected to ensure that the observed dissolution behavior reflects intrinsic polymer/API interactions rather than loading-dependent phase separation effects.

The dissolution of carbamazepine (Figure 9A) was enhanced in KVA64, RLPO and EPO ASDs, but significantly reduced in HPMCAS relative to the neat API. FTIR spectra of ASDs prepared with EPO and RLPO (Figure 5A,B) did not show evidence of strong specific interactions. Contrarily, ASDs prepared with HPMCAS (Figure 5C) exhibited clear evidence of strong intermolecular interactions, likely arising from hydrogen bonding or ion pairs.^28^ Despite exhibiting plasticization behavior (Figure 8A), these interactions appear to restrict API release. This observation is consistent with prior reports for lipophilic, weakly basic APIs in HPMCAS systems, where ionic interactions and electrostatic complexation reduce aqueous solubility and dissolution performance.^42^

**Figure 9:**
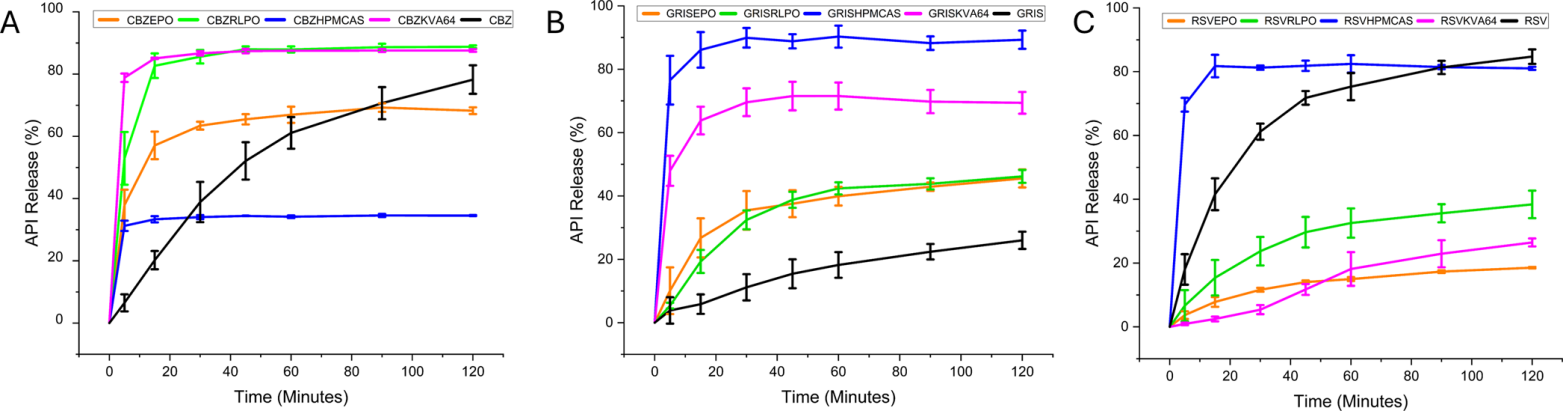
Dissolution plots showing API release of (A) carbamazepine, (B) griseofulvin, and (C) resveratrol from polymer matrices.

With respect to griseofulvin, amorphization yielded a substantial solubility enhancement for dissolution (Figure 9B) relative to the crystalline API. Due to minimal intermolecular interactions, KVA64 and HPMCAS improve solubility by 132% and 235% respectively. Additionally, HPMCAS is plasticized by GRIS (Figure 8B), suggesting increased molecular mobility that can facilitate faster dissolution. EPO and RLPO, which show stronger specific interactions by FTIR (Figure 6A,B) and are antiplasticized by the API (Figure 8B), only improve API release by 55% compared to crystalline GRIS.

A similar trend was observed with RSV. In ASD systems where RSV acts as an antiplasticizer (EPO, RLPO, and KVA64), API release was limited due to reduced polymer chain mobility. Conversely, in HPMCAS where RSV shows plasticization by DSC (Figure 8C), a burst release was observed, whereby 73% release within the first 15 min of dissolution was obtained.

## Conclusion

In this work, we demonstrated that experimentally determined Hansen solubility parameters (HSP) provide a reliable framework for predicting polymer/API miscibility in ASD formulations, outperforming conventional group contribution methods. Across the systems evaluated, miscibility predictions based on experimentally derived HSP values exhibited a strong correlation with API loading capacity, confirming their utility as a formulation design tool. Hot melt extrusion was performed with materials sparing, where only 2–7 g of API was used to determine the maximum amorphous API soluble in each matrix. While RED values were effective for identifying the MADS of polymer/API systems, relying on the magnitude of the RED alone is not sufficient to fully describe formulation performance.

Polymer/API interactions, as evidenced by peak shifts in FTIR and deviations from ideal mixing behavior in glass transition temperature, were observed across both highly and poorly miscible systems. Across these ASD combinations, dissolution behavior was governed by a balance between interaction strength and polymer matrix mobility. Most systems exhibiting negative deviations from ideal mixing behavior display enhanced API dissolution, attributed to increased polymer chain mobility. In these cases, burst release was achieved for all three BCS class II APIs evaluated, with over 80% of the API released within the first 30 min of dissolution.

Overall, this work highlights the importance of a comprehensive approach to ASD design that integrates experimentally derived Hansen solubility parameter analysis with complementary physicochemical characterization techniques. By improving our understanding of miscibility prediction, polymer/API interactions, and polymer chain mobility, more stable, high API-loaded ASDs with optimized performance can be rationally designed.

## Supplementary Material



## Notes

HSP scoring criteria for polymers and APIs, HPLC conditions for API detection, TGA thermograms of ASDs, polarized optical micrographs of griseofulvin loaded ASDs and neat HPMCAS, true densities and coefficient of thermal expansivity of materials for Gordon-Taylor calculations, DSC thermograms of APIs, polymers, and ASDs, XRD showing of ASDs at MADS concentration and 1 year stability study, and FTIR spectra of (A) CBZKVA64, (B) GRISKVA64, and (C) RSVRLPO (PDF)

## References

[1] Ting J. M., Porter W. W., Mecca J. M., Bates F. S., Reineke T. M. (2018). Advances
in Polymer Design for Enhancing Oral Drug Solubility and Delivery. Bioconjugate Chem..

[2] Hancock B. C., Zografi G. (1997). Characteristics and
Significance of the Amorphous State
in Pharmaceutical Systems. J. Pharm. Sci..

[3] Shah, N. ; Sandhu, N. ; Choi, D. S. ; Chokshi, H. ; Waseem Malick, A. Amorphous Solid Dispersions Theory and Practice; Springer: New York, 2014.

[4] Taylor L. S., Zografi G. (2025). From Unwanted Annoyances to Oral
Delivery Saviors:
The Rollercoaster Journey of Amorphous Drugs. Mol. Pharmaceutics.

[5] Mamidi H., Palekar S., Patel H., Nukala P. K., Patel K. (2023). Formulation
strategies for the development of high drug-loaded amorphous solid
dispersions. Drug Discovery Today.

[6] Farrell B., French Merkley V., Ingar N. (2013). Reducing pill burden and helping
with medication awareness to improve adherence. Can. Pharm. J..

[7] Singh D., Krishna V., Kumari N., Banerjee A., Kapoor P. (2024). Thermodynamics
of Polymer Drug Interactions: An Influential Factor for the Development
of a Stable Drug Delivery System. J. Macromol.
Sci., Part B:Phys..

[8] Yani Y., Kanaujia P., Chow P. S., Tan R. B. H. (2017). Effect of API-Polymer
Miscibility and Interaction on the Stabilization of Amorphous Solid
Dispersion: A Molecular Simulation Study. Ind.
Eng. Chem. Res..

[9] Thakral S., Thakral N. K. (2013). Prediction of Drug–Polymer
Miscibility through
the use of Solubility Parameter based Flory–Huggins Interaction
Parameter and the Experimental Validation: PEG as Model Polymer. J. Pharm. Sci..

[10] Gani R. (2019). Group contribution-based
property estimation methods: advances and perspectives. Curr. Opin. Chem. Eng..

[11] Marsac P. J., Li T., Taylor L. S. (2009). Estimation
of Drug–Polymer Miscibility and Solubility
in Amorphous Solid Dispersions Using Experimentally Determined Interaction
Parameters. Pharm. Res..

[12] Turpin E. R., Taresco V., Al-Hachami W. A., Booth J., Treacher K., Tomasi S., Alexander C., Burley J., Laughton C. A., Garnett M. C. (2018). In Silico Screening
for Solid Dispersions: The Trouble
with Solubility Parameters and χFH. Mol.
Pharmaceutics.

[13] Leroy F. (2013). Molecular
Driving Forces. Statistical Thermodynamics in Biology, Chemistry,
Physics, and Nanoscience. Soft Mater..

[14] He Y., Ho C. (2015). Amorphous Solid Dispersions:
Utilization and Challenges in Drug Discovery
and Development. J. Pharm. Sci..

[15] Ajjarapu S., Banda S., Basim P., Dudhipala N. (2022). Melt Fusion
Techniques for Solubility Enhancement: A Comparison of Hot Melt Extrusion
and KinetiSol Technologies. Sci. Pharm..

[16] Huang S., Williams R. O. (2018). Effects of the Preparation Process
on the Properties
of Amorphous Solid Dispersions. AAPS PharmSciTech.

[17] Nikam A., Sahoo P. R., Musale S., Pagar R. R., Paiva-Santos A. C., Giram P. S. (2023). A Systematic Overview
of Eudragit Based Copolymer for
Smart Healthcare. Pharmaceutics.

[18] Patel K. G., Maynard R. K., Ferguson L. S., Broich M. L., Bledsoe J. C., Wood C. C., Crane G. H., Bramhall J. A., Rust J. M., Williams-Rhaesa A., Locklin J. J. (2024). Experimentally Determined
Hansen
Solubility Parameters of Biobased and Biodegradable Polyesters. ACS Sustainable Chem. Eng..

[19] Zellers E. T., Anna D. H., Sulewski R., Wei X. (1996). Critical analysis
of
the graphical determination of hansen’s solubility parameters
for lightly crosslinked polymers. J. Appl. Polym.
Sci..

[20] Abbott, S. ; Hansen, C. M. Hansen Solubility Parameters in Practice; Hansen-Solubility, 2020.

[21] Hansen, C. M. Hansen Solubility Parameters: a User’s Handbook; CRC Press, 2007.

[22] Stefanis E., Panayiotou C. (2008). Prediction of Hansen Solubility Parameters with a New
Group-Contribution Method. Int. J. Thermophys..

[23] Li Z., Constantinou L., Baur R., Dubbeldam D., Calero S., Sharma S., Rigutto M., Dey P., Vlugt T. J. H. (2025). Review of group
contribution methods for prediction
of thermodynamic properties of long-chain hydrocarbons. Mol. Phys..

[24] Chang C., Lu C., Zheng Y., Ji J., Lin L., Chen L., Chen Z., Chen R. (2024). Sonication-Assisted Self-Assembled
Resveratrol Nanoparticles with Enhanced Antiviral and Anti-inflammatory
Activity against Respiratory Syncytial Virus-Induced Pneumonia. ACS Appl. Mater. Interfaces.

[25] Wegiel L. A., Mauer L. J., Edgar K. J., Taylor L. S. (2013). Crystallization
of Amorphous Solid Dispersions of Resveratrol during Preparation and
Storage-Impact of Different Polymers. J. Pharm.
Sci..

[26] Al-Obaidi H., Buckton G. (2009). Evaluation of Griseofulvin
Binary and Ternary Solid
Dispersions with HPMCAS. AAPS PharmSciTech.

[27] Rahman M., Coelho A., Tarabokija J., Ahmad S., Radgman K., Bilgili E. (2020). Synergistic and antagonistic
effects of various amphiphilic
polymer combinations in enhancing griseofulvin release from ternary
amorphous solid dispersions. Eur. J. Pharm.
Sci..

[28] Ishizuka Y., Ueda K., Okada H., Takeda J., Karashima M., Yazawa K., Higashi K., Kawakami K., Ikeda Y., Moribe K. (2019). Effect of Drug–Polymer
Interactions through
Hypromellose Acetate Succinate Substituents on the Physical Stability
on Solid Dispersions Studied by Fourier-Transform Infrared and Solid-State
Nuclear Magnetic Resonance. Mol. Pharmaceutics.

[29] Bennett R. C., Keen J. M., Bi Y., Porter S., Dürig T., McGinity J. W. (2015). Investigation of
the interactions of enteric and hydrophilic
polymers to enhance dissolution of griseofulvin following hot melt
extrusion processing. J. Pharm. Pharmacol..

[30] Lu J., Obara S., Liu F., Fu W., Zhang W., Kikuchi S. (2018). Melt Extrusion for a High Melting
Point Compound with
Improved Solubility and Sustained Release. AAPS
PharmSciTech.

[31] Wu H., Liu Y., Ci T., Ke X. (2020). Application of HPMC HME polymer as
hot melt extrusion carrier in carbamazepine solid dispersion. Drug Dev. Ind. Pharm..

[32] Alshahrani S. M., Lu W., Park J.-B., Morott J. T., Alsulays B. B., Majumdar S., Langley N., Kolter K., Gryczke A., Repka M. A. (2015). Stability-enhanced
Hot-melt Extruded Amorphous Solid Dispersions via Combinations of
Soluplus and HPMCAS-HF. AAPS PharmSciTech.

[33] Liu J., Cao F., Zhang C., Ping Q. (2013). Use of polymer combinations in the
preparation of solid dispersions of a thermally unstable drug by hot-melt
extrusion. Acta Pharm. Sin. B.

[34] Zhou H., Wang Y., Li S., Lu M. (2021). Improving chemical
stability of resveratrol in hot melt extrusion based on formation
of eutectic with nicotinamide. Int. J. Pharm..

[35] Wegiel L. A., Mosquera-Giraldo L. I., Mauer L. J., Edgar K. J., Taylor L. S. (2015). Phase Behavior
of Resveratrol Solid Dispersions Upon Addition to Aqueous media. Pharm. Res..

[36] Kumar A., Singh P., Nanda A. (2020). Hot stage microscopy
and its applications
in pharmaceutical characterization. Appl. Microsc..

[37] Rustichelli C., Gamberini G., Ferioli V., Gamberini M. C., Ficarra R., Tommasini S. (2000). Solid-state
study of polymorphic
drugs: carbamazepine. J. Pharm. Biomed. Anal..

[38] Baird J. A., Van Eerdenbrugh B., Taylor L. S. (2010). A Classification System to Assess
the Crystallization Tendency of Organic Molecules from Undercooled
Melts. J. Pharm. Sci..

[39] Alhalaweh A., Alzghoul A., Kaialy W., Mahlin D., Bergström C. A. S. (2014). Computational
Predictions of Glass-Forming Ability and Crystallization Tendency
of Drug Molecules. Mol. Pharmaceutics.

[40] Shi Q., Tao J., Zhang J., Su Y., Cai T. (2020). Crack- and Bubble-Induced
Fast Crystal Growth of Amorphous Griseofulvin. Cryst. Growth Des..

[41] Zhou D., Zhang G. G. Z., Law D., Grant D. J. W., Schmitt E. A. (2008). Thermodynamics
Molecular Mobility and Crystallization Kinetics of Amorphous Griseofulvin. Mol. Pharmaceutics.

[42] Bapat P., Paul S., Tseng Y.-C., Taylor L. S. (2024). Interplay of Drug–Polymer
Interactions and Release Performance for HPMCAS-Based Amorphous Solid
Dispersions. Mol. Pharmaceutics.

[43] Saboo S., Kestur U. S., Flaherty D. P., Taylor L. S. (2020). Congruent Release
of Drug and Polymer from Amorphous Solid Dispersions: Insights into
the Role of Drug-Polymer Hydrogen Bonding, Surface Crystallization,
and Glass Transition. Mol. Pharmaceutics.

[44] Kothari K., Ragoonanan V., Suryanarayanan R. (2015). The Role of Drug–Polymer Hydrogen
Bonding Interactions on the Molecular Mobility and Physical Stability
of Nifedipine Solid Dispersions. Mol. Pharmaceutics.

[45] Deac A., Qi Q., Indulkar A. S., Purohit H. S., Gao Y., Zhang G. G. Z., Taylor L. S. (2023). Dissolution
Mechanisms of Amorphous Solid Dispersions:
Role of Drug Load and Molecular Interactions. Mol. Pharmaceutics.

[46] Feng Z., Li M., Wang W. (2019). Improvement
of dissolution and tabletability of carbamazepine
solid dispersions with high drug loading prepared by hot-melt extrusion. Pharmazie.

[47] Yu D., Li J., Wang H., Pan H., Li T., Bu T., Zhou W., Zhang X. (2022). Role of polymers in the physical
and chemical stability of amorphous solid dispersion: A case study
of carbamazepine. Eur. J. Pharm. Sci..

[48] Benmore C. J., Edwards A., Alderman O. L. G., Cherry B. R., Smith P., Smith D., Byrn S., Weber R., Yarger J. L. (2022). The Structure
of Liquid and Glassy Carbamazepine. Quantum
Beam Sci..

[49] Mah P. T., Novakovic D., Saarinen J., Van Landeghem S., Peltonen L., Laaksonen T., Isomäki A., Strachan C. J. (2017). Elucidation of Compression-Induced
Surface Crystallization
in Amorphous Tablets Using Sum Frequency Generation (SFG) Microscopy. Pharm. Res..

[50] Al-Obaidi H., Brocchini S., Buckton G. (2009). Anomalous properties
of spray dried
solid dispersions. J. Pharm. Sci..

[51] Garai J. (2006). Correlation
between thermal expansion and heat capacity. Calphad.

[52] Van
den Mooter G., Wuyts M., Blaton N., Busson R., Grobet P., Augustijns P., Kinget R. (2001). Physical stabilisation
of amorphous ketoconazole in solid dispersions with polyvinylpyrrolidone
K25. Eur. J. Pharm. Sci..

[53] Forster A., Hempenstall J., Tucker I., Rades T. (2001). The Potential of Small-Scale
Fusion Experiments and the Gordon-Taylor Equation to Predict the Suitability
of Drug/Polymer Blends for Melt Extrusion. Drug
Dev. Ind. Pharm..

[54] Indulkar A. S., Lou X., Zhang G. G. Z., Taylor L. S. (2019). Insights into the Dissolution Mechanism
of Ritonavir–Copovidone Amorphous Solid Dispersions: Importance
of Congruent Release for Enhanced Performance. Mol. Pharmaceutics.

[55] Fahrig I., Walter S., Kyeremateng S., Degenhardt M., Sadowski G., Brandenbusch C. (2025). The More the
Better?–Vitamin
E TPGS as a Release Enhancer for Ritonavir/PVPVA Amorphous Solid Dispersions. Mol. Pharmaceutics.

[56] Correa
Soto C. E., Gao Y., Indulkar A. S., Ueda K., Zhang G. G. Z., Taylor L. S. (2022). Impact of Surfactants on the Performance
of Clopidogrel-Copovidone Amorphous Solid Dispersions: Increased Drug
Loading and Stabilization of Nanodroplets. Pharm.
Res..

