# Supplementary material for: Experimentally Derived Hansen Solubility Parameters as a Screening Tool for the Formulation of Amorphous Solid Dispersions
Source: ACS Omega. 2026 Jun 22;11(26):39417–28. doi: 10.1021/acsomega.6c05058 (PMC13347368; doi:10.1021/acsomega.6c05058)
Supplement: Supplementary file 1 [file ao6c05058_si_001.pdf]

Supporting Information

Experimentally Derived Hansen Solubility Parameters as a Screening Tool for the Formulation of Amorphous Solid Dispersions

Authors – Adaeze R. Osakwe [‡, †], Mira T.N. Le [‡, †], Jessica A. Bramhall [†], Vladislav V. Klepov [‡], and Jason J. Locklin [‡, †, *]

‡ Department of Chemistry, Franklin College of Arts and Sciences, University of Georgia, 140 Cedar Street, Athens, Georgia, 30602, United States.

† New Materials Institute, University of Georgia, 220 Riverbend Road, Athens, Georgia, 30602, United States.

* Corresponding Author – email: jlocklin@uga.edu

Table of Contents

Table S1. HSP scores assigned to solvents for each polymer according to the criteria defined in Table S2.

Table S2. HSP scoring criteria for each polymer and the APIs.

Table S3. HPLC Methods.

Figure S1. Thermogravimetric analysis of carbamazepine ASDs formed with a) EPO, b) RLPO, c) HPMCAS, and d) KVA64.

Figure S2. Thermogravimetric analysis of griseofulvin ASDs formed with a) EPO, b) RLPO, c) HPMCAS, and d) KVA64.

Figure S3. Thermogravimetric analysis of resveratrol ASDs formed with a) EPO, b) RLPO, c) HPMCAS, and d) KVA64.

Figure S4. Polarized optical micrographs of physical mixtures of griseofulvin – a) 10% GRISEPO b) 20% GRISEPO c) 10% GRISRLPO d) 20% GRISRLPO e) 10% GRISKVA64 f) 20% GRISKVA64.

Table S4. True densities and co-efficient of thermal expansivity of materials.

Figure S5. First heating curves of DSC thermograms showing glass transition temperatures of amorphous carbamazepine and griseofulvin.

Figure S6. Second heating curves of DSC thermograms showing glass transition temperatures of carbamazepine ASDs formed with a) EPO, b) RLPO, c) HPMCAS, and d) KVA64.

Figure S7. Second heating curves of DSC thermograms showing glass transition temperatures of griseofulvin ASDs formed with a) EPO, b) RLPO, c) HPMCAS, and d) KVA64.

Figure S8. Second heating curves of DSC thermograms showing glass transition temperatures of resveratrol ASDs formed with a) EPO, b) RLPO, c) HPMCAS, and d) KVA64

Figure S9. X-ray diffractograms of amorphous solid dispersions (ASDs) at MADS containing (a) carbamazepine, (b) griseofulvin, and (c) resveratrol, after one year of storage under ambient conditions

Figure S10. FTIR spectra of A) CBZKVA64, B) GRISKVA64, and C) RSVRLPO

Table S1. Solvent score assignments for each polymer and API according to the criteria defined in Table S2.

Solvent	Eu-EPO	Eu-RLPO	HPC	HPMC	AS-H	AS-M	AS-L	KVA 64	PVP K30	Solu plus	CBZ	GRIS	RSV
**Acetonitrile**	-	-	5	5	-	1	4	1	-	1	1	1	1
**Tetrahydrofuran**	1	1	4	4	1	1	1	1	1	1	1	1	1
**Toluene**	-	4	6	6	5	6	3	4	4	1	6	6	6
**Ethyl Acetate**	1	1	5	5	1	1	1	1	5	1	2	2	1
**DCM**	1	1	4	1	-	4	-	1	1	1	1	1	4
**Chloroform**	1	1	4	1	1	4	4	1	1	1	1	1	6
**Hexane**	6	6	6	6	6	6	6	6	6	6	-	-	-
**Dimethyl Aniline**	1	1	6	6	4	4	4	4	4	4	-	-	6
**2-Propanol**	1	4	4	5	6	6	5	1	1	2	4	6	1
**2-Pyrrolidinone**	-	1	1	1	1	1	2	-	1	1	-	-	-
**Acetone**	1	1	4	5	1	1	1	1	1	1	1	1	1
**DMSO**	6	1	1	-	2	1	2	1	1	1	1	1	1
**Dimethyl Acetamide**	1	1	1	1	1	1	2	1	1	1	-	-	-
**Carbon Tetrachloride**	-	5	6	6	6	6	6	5	6	6	-	-	4
**Propylene Carbonate**	2	-	6	6	2	1	-	-	-	2	-	-	-
**Dipropylene Glycol**	-	-	4	6	4	4	4	2	1	3	-	-	-
**Pyridine**	1	1	1	1	1	1	2	1	1	1	1	1	-
**Ethanol**	-	-	4	4	5	5	4	1	1	-	1	3	4
**Triethyl Amine**	2	6	6	6	6	6	6	6	4	6	-	-	-
**Ethanolamine**	-	6	-	-	2	-	-	2	-	4	-	-	-
**Styrene**	1	2	-	-	5	5	5	-	-	5	6	3	-
**Diethyl ether**	1	5	-	-	-	-	-	6	-	5	6	6	6
**Cresol**	1	1	-	-	2	2	2	-	-	-	-	-	-
**Aniline**	1	-	-	-	-	-	-	-	-	-	1	1	-
**Cyclohexane**	6	-	-	-	-	-	-	6	-	6	6	6	6
**Tetrahydronaphthalene**	1	-	-	6	-	-	-	-	-	-	6	4	6
**Ethylene Glycol**	6	-	-	5	-	-	-	-	-	-	-	-	-
**Diethyl Sulfide**	1	-	-	-	-	--	-	-	-	-	-	-	-
**Furan**	1	-	-	5	-	-	-	-	-	-	-	-	-
**1, 4- dioxane**	1	-	-	1	-	-	-	-	-	-	1	1	-
**Dimethoxy ethane**	1	-	4	4	-	-	-	-	-	-	-	-	-
**Methyl Ethyl Ketone**	-	-	4	5	1	1	1	-	-	-	1	1	1
**Chlorobenzene**	-	-	-	-	-	-	-	-	-	-	3	2	6
**HFIP**	-	-	-	-	-	-	-	-	-	-	1	1	4
**Xylene**	-	-	-	-	-	-	-	-	-	-	-	-	6
**Ethyl lactate**	-	-	-	1	1	1	1	-	-	-	-	-	-
**Total Solvents**	24	19	21	25	22	23	21	20	17	22	20	20	19

Table S2. HSP scoring criteria for each polymer and the APIs.

Where: D1hr – Dissolved in 1 hour, D24hr – Dissolved in 24 hours, D4dy – Dissolved in 4 days, UB – unrecoverable blob, 1000-100 – Normalized solvent uptake $\left(\frac{\mu L}{g}\right)$, and N.D – Not dissolved at 3 ml

Score	Eu-EPO	Eu-RLPO	HPC	HPMC	HPMCAS-H	HPMCAS-M	HPMCAS-L	KVA 64	PVP K30	Soluplus	APIs
1	D1hr	D1hr	D1hr	D1hr	D1hr	D1hr	D1hr	D1hr	D1hr	D1hr	0.5 ml
2	D24hr	D24hr	D24hr	D24hr	D24hr	D24hr	D24hr	D24hr	D24hr	D24hr	1 ml
3	D4dy	D4dy	D4dy	D4dy	D4dy	D4dy	D4dy	D4dy	D4dy	D4dy	1.5 ml
4	UB	UB	UB	1000-300	UB	UB	UB	UB	1000-500	UB	2 ml
5	1000-500	1000-500	1000-500	300-100	1000-500	1000-500	1000-500	1000-200	500-200	1000-500	2.5 – 3ml
6	<500	<500	<500	<100	<500	<500	<500	<200	<200	<500	N.D

Table S3. HPLC Methods.

Chromatographic Parameters	Carbamazepine	Griseofulvin	Resveratrol
Mobile Phase A	0.05% triethylamine in water (pH 7.3, neutralized with H_3PO_4)	55% 20mM NaH_2PO_4 (pH 3.5, acidified with H_3PO_4)	70% 10mM NH_4Cl (pH 4, acidified with HCl)
Mobile Phase B	35% Acetonitrile	45% Acetonitrile	30% Acetonitrile
Flow rate	1 ml/min	1.0 mL/min	0.9 mL/min
Injection volume	10 μL	50 μL	10 μL
Column temperature	25 °C	25 °C	25 °C
Detection wavelength	230 nm	291 nm	250 nm
Elution time	4.7 minutes	5.8 minutes	4.9 minutes

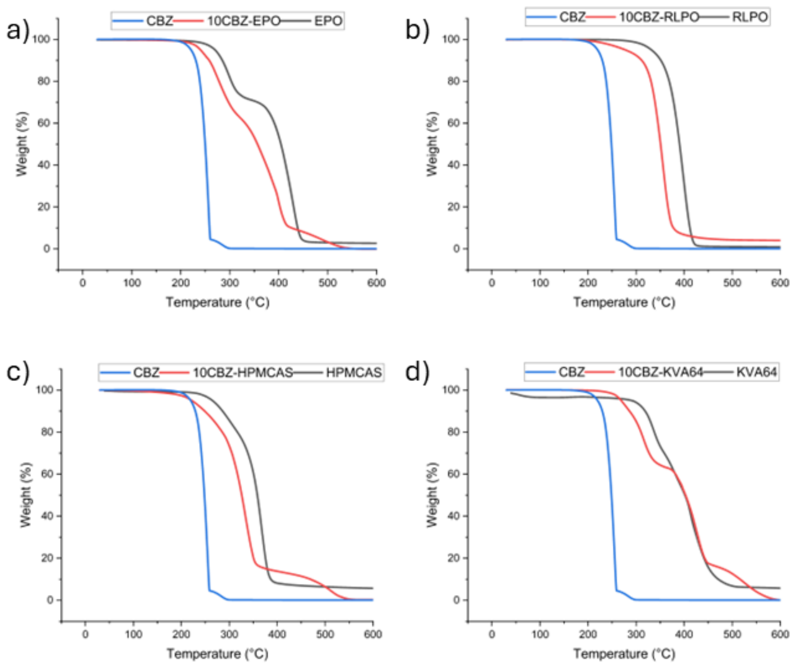

Figure S1. Thermogravimetric analysis of carbamazepine ASDs formed with a) EPO, b) RLPO, c) HPMCAS, and d) KVA64.

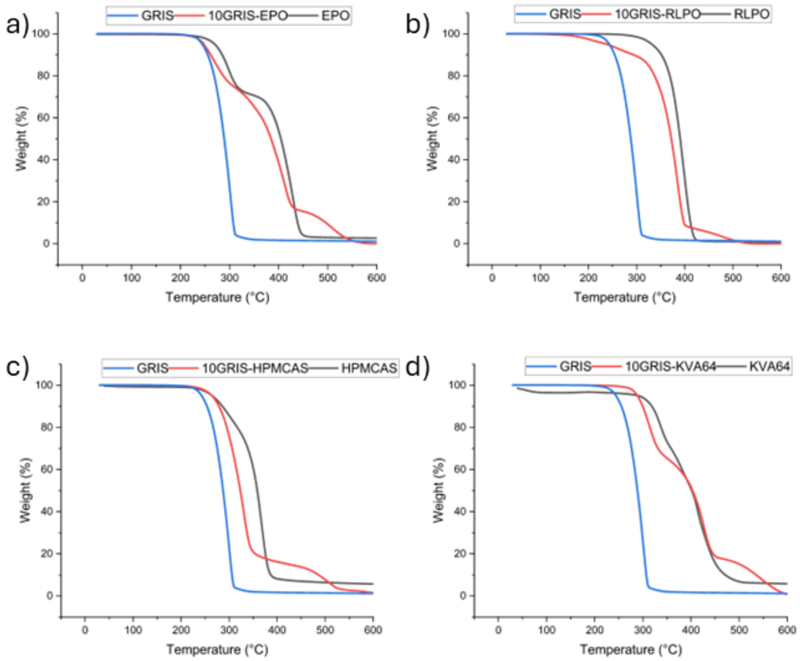

Figure S2. Thermogravimetric analysis of griseofulvin ASDs formed with a) EPO, b) RLPO, c) HPMCAS, and d) KVA64.

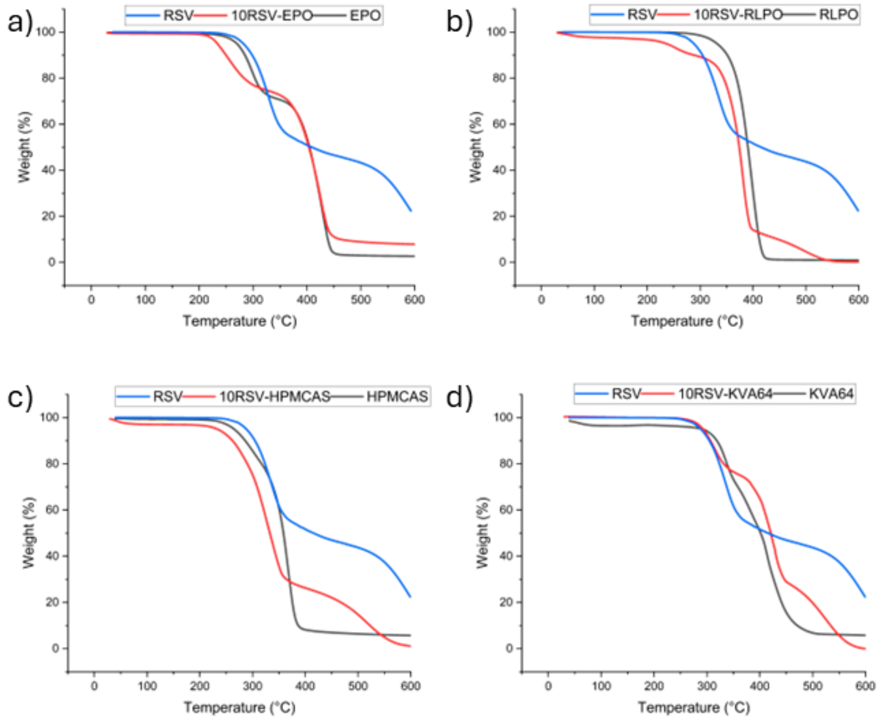

Figure S3. Thermogravimetric analysis of resveratrol ASDs formed with a) EPO, b) RLPO, c) HPMCAS, and d) KVA64.

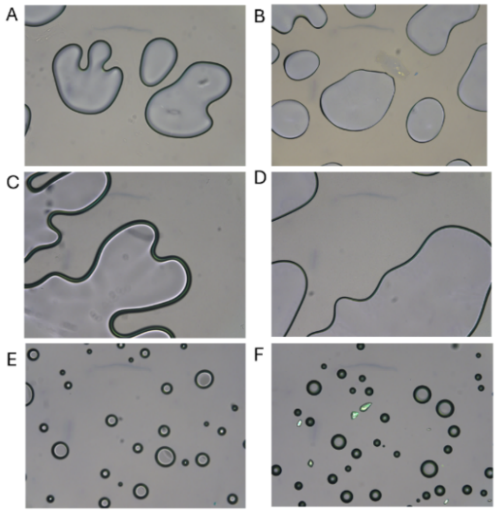

i)

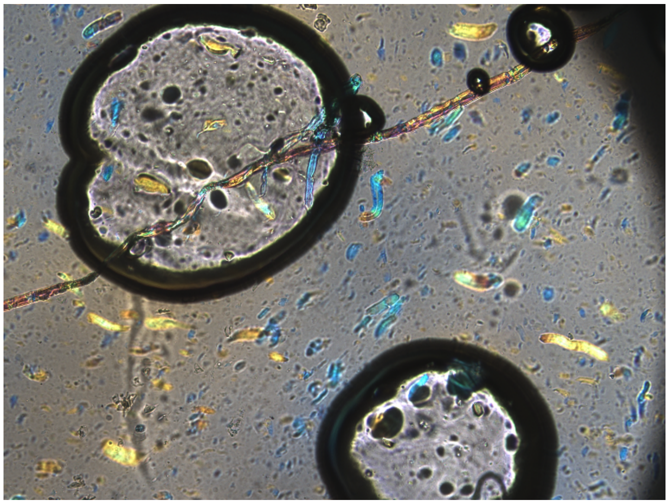

ii)

Figure S4. Polarized optical micrographs of: i) Physical mixtures of griseofulvin – a) 10% GRISEPO b) 20% GRISEPO c) 10% GRISRLPO d) 20% GRISRLPO e) 10% GRISKVA64 f) 20% GRISKVA64. ii) Neat HPMCAS show presence of crystallites (blue and yellow particles). Black circles are due to formation of air bubbles trapped between glass sides.

Table S4. True densities and co-efficient of thermal expansivity.

**Material**	**True Density (g/ml)**	**Coefficient of thermal expansion (K^{-1})** [a]
Carbamazepine [1]	1.34	1.3 x10^{-4}
Griseofulvin [2]	1.489	1.79 x10^{-4}
Resveratrol	1.4011	1.2 x10^{-4}
Eudragit EPO	1.2224	1
Eudragit RLPO	1.1926	0.837
HPMCAS LMP	1.2819	0.806
Kollidon VA 64	1.2121	0.671

a. Values obtained for APIs from literature or modulated DSC correlation for polymers.

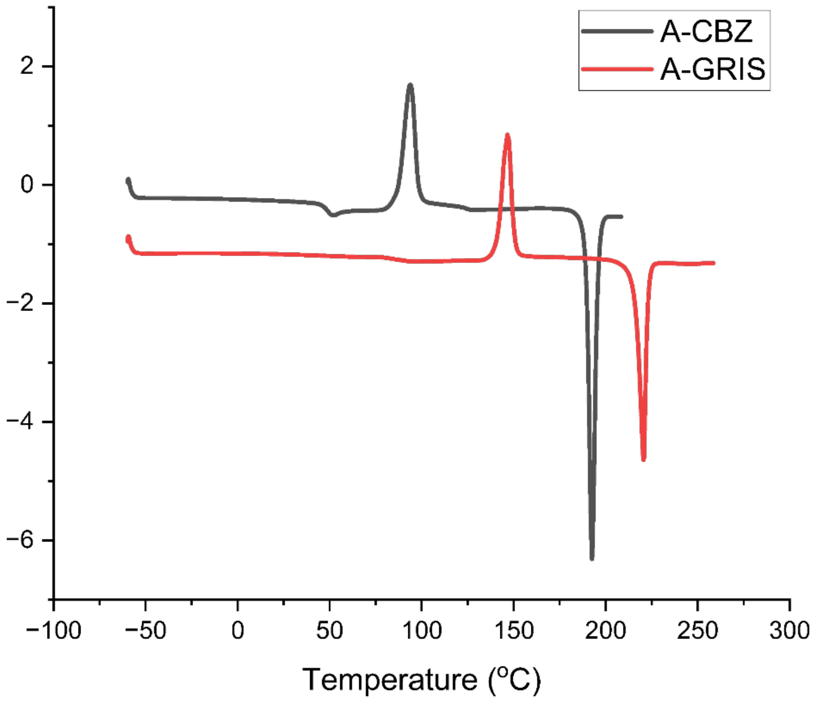

Figure S5. First heating curves of DSC thermograms showing glass transition temperatures of amorphous carbamazepine and griseofulvin.

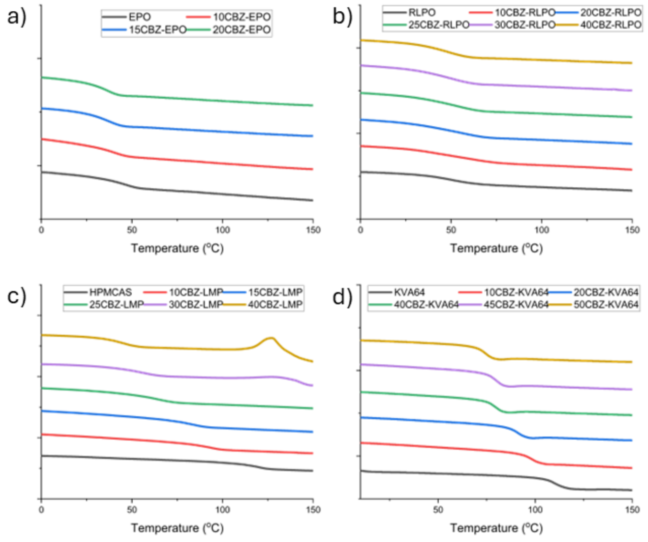

Figure S6. Second heating curves of DSC thermograms showing glass transition temperatures of carbamazepine ASDs formed with a) EPO, b) RLPO, c) HPMCAS, and d) KVA64.

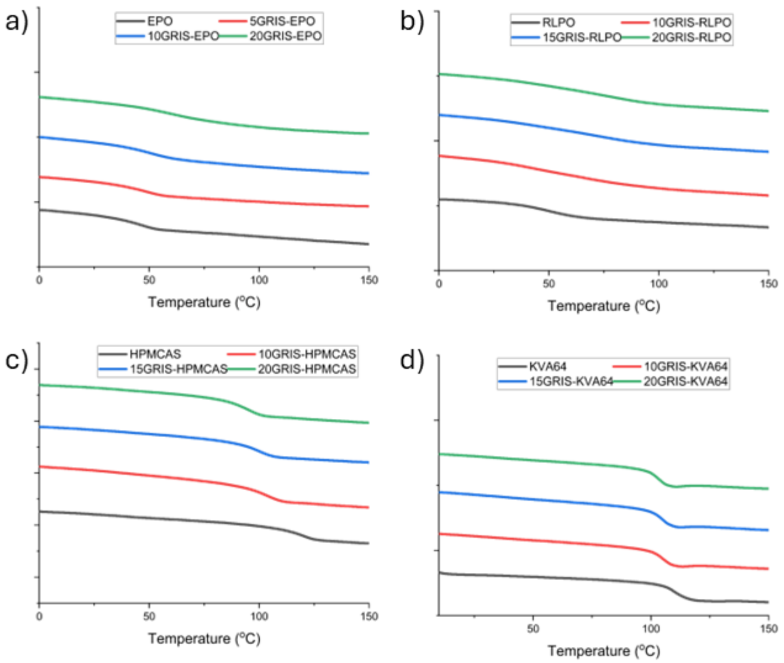

Figure S7. Second heating curves of DSC thermograms showing glass transition temperatures of griseofulvin ASDs formed with a) EPO, b) RLPO, c) HPMCAS, and d) KVA64.

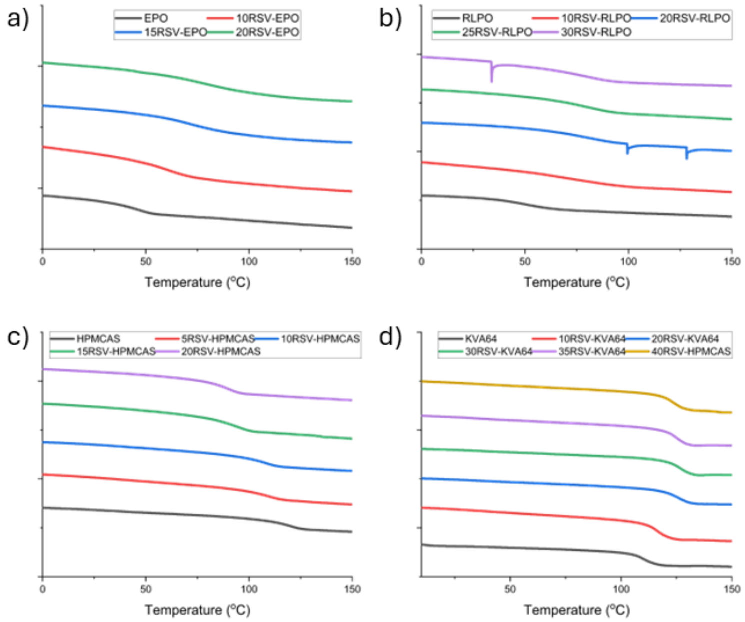

Figure S8. Second heating curves of DSC thermograms showing glass transition temperatures of resveratrol ASDs formed with a) EPO, b) RLPO, c) HPMCAS, and d) KVA64.

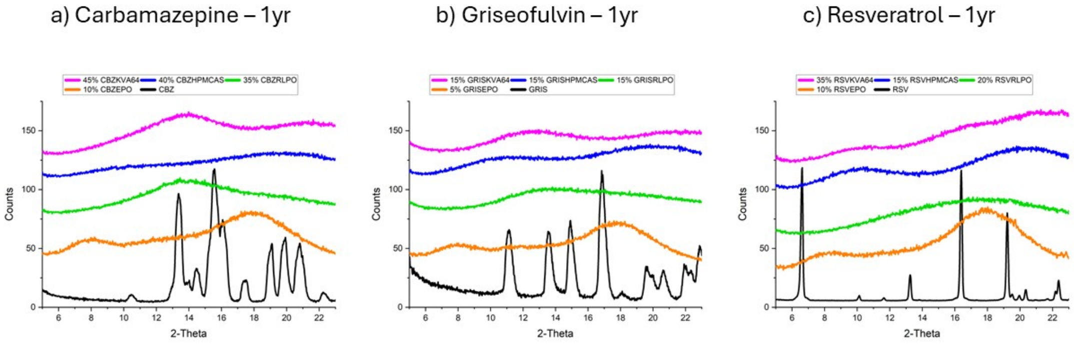

Figure S9. X-ray diffractograms of amorphous solid dispersions (ASDs) at MADS containing (a) carbamazepine, (b) griseofulvin, and (c) resveratrol, after one year of storage under ambient conditions.

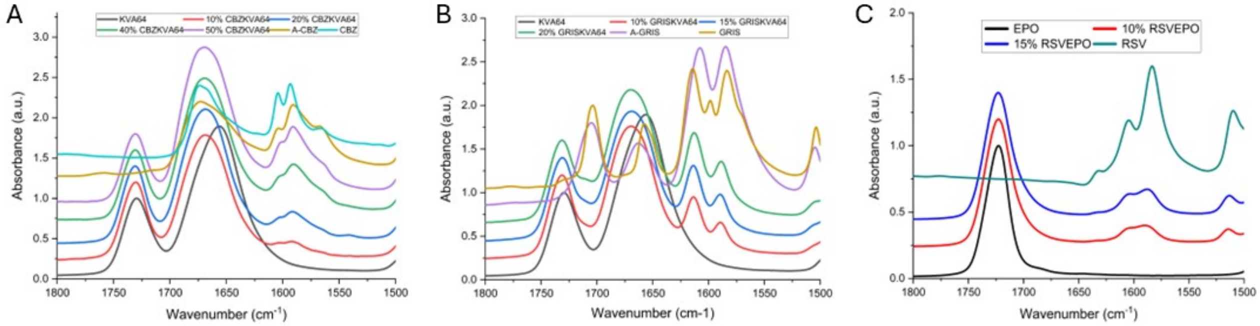

Figure S10. FTIR spectra of A) CBZKVA64, B) GRISKVA64, and C) RSVEPO.

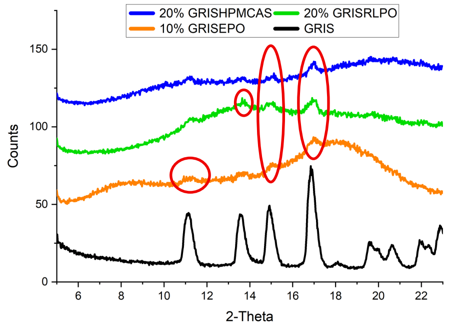

Figure S11. X-ray diffractograms of griseofulvin (black) and its solid dispersions above the MADS concentration in EPO (orange), RLPO (green), and HPMCAS (blue).

References

(1) Nicolaï, B.; Fournier, B.; Dahaoui, S.; Gillet, J.-M.; Ghermani, N.-E. Crystal and Electron Properties of Carbamazepine–Aspirin Co-crystal. *Crystal Growth & Design* **2018**, *19* (2), 1308-1321. DOI: 10.1021/acs.cgd.8b01698.
(2) Su, Y.; Xu, J.; Shi, Q.; Yu, L.; Cai, T. Polymorphism of griseofulvin: concomitant crystallization from the melt and a single crystal structure of a metastable polymorph with anomalously large thermal expansion. *Chemical Communications* **2018**, *54* (4), 358-361. DOI: 10.1039/c7cc07744k.
